# Retrospective Single-Center Case Study of Clinical Variables and the Degree of Actinic Elastosis Associated with Rare Skin Cancers

**DOI:** 10.3390/biology13070529

**Published:** 2024-07-16

**Authors:** Konstantin Drexler, Lara Bollmann, Sigrid Karrer, Mark Berneburg, Sebastian Haferkamp, Dennis Niebel

**Affiliations:** Department of Dermatology, University Medical Center Regensburg, 93053 Regensburg, Germany; konstantin.drexler@ukr.de (K.D.);

**Keywords:** atypical fibroxanthoma, pleomorphic dermal sarcoma, dermatofibrosarcoma protuberans, Merkel cell carcinoma, Kaposi sarcoma, leiomyosarcoma

## Abstract

**Simple Summary:**

While sun exposure and associated tissue changes stemming from ultraviolet radiation are closely associated with the most common forms of skin cancer, far less is known regarding rare types of skin cancer. In this study, for the first time, we used a light microscopy technique to evaluate connective tissue changes in samples from patients with six different types of rare skin cancers, assessing the relationship between these changes, patient age, and whether tumors arose on sun-exposed parts of the body. We found that these tissue changes were most pronounced for patients with specific cancers known to be linked to chronic sun damage and tumors arising on sun-exposed parts of the body. We also noted tumor type-specific trends in terms of sex ratios, sites of tumor presentation, and the relationship between the development of particular tumors and patient immunosuppression. Our results are important and novel as they expand the available data associated with these rare skin cancers while also offering insight into the value of differentiating among these tumor types based on their relationship with sun exposure, potentially informing preventative, diagnostic, and/or therapeutic approaches.

**Abstract:**

(1) Background: Rare skin cancers include epithelial, neuroendocrine, and hematopoietic neoplasias as well as cutaneous sarcomas. Ultraviolet (UV) radiation and sunburns are important drivers for the incidence of certain cutaneous sarcomas; however, the pathogenetic role of UV light is less clear in rare skin cancers compared to keratinocyte cancer and melanoma. In this study, we compared the degree of actinic elastosis (AE) as a surrogate for lifetime UV exposure among selected rare skin cancers (atypical fibroxanthoma [AFX], pleomorphic dermal sarcoma [PDS], dermatofibrosarcoma protuberans [DFSP], Kaposi sarcoma [KS], Merkel cell carcinoma [MCC], and leiomyosarcoma [LMS]) while taking into account relevant clinical variables (age, sex, and body site). (2) Methods: We newly established a semi-quantitative score for the degree of AE ranging from 0 = none to 3 = total loss of elastic fibers (basophilic degeneration) and multiplied it by the perilesional vertical extent (depth), measured histometrically (tumor-associated elastosis grade (TEG)). We matched the TEG of *n* = 210 rare skin cancers from 210 patients with their clinical variables. (3) Results: TEG values were correlated with age and whether tumors arose on UV-exposed body sites. TEG values were significantly higher in AFX and PDS cases compared to all other analyzed rare skin cancer types. As expected, TEG values were low in DFSP and KS, while MCC cases exhibited intermediate TEG values. (4) Conclusions: High cumulative UV exposure is more strongly associated with AFX/PDS and MCC than with other rare skin cancers. These important results expand the available data associated with rare skin cancers while also offering insight into the value of differentiating among these tumor types based on their relationship with sun exposure, potentially informing preventative, diagnostic and/or therapeutic approaches.

## 1. Introduction

Cutaneous malignancies remain the most common type of cancer, with the vast majority comprising basal cell carcinoma (BCC), squamous cell carcinoma (SCC), and cutaneous melanoma cases that have been characterized extensively [1]. In contrast, up to 5% of non-melanoma skin cancers consist of rare entities arising from neuroendocrine, mesenchymal, vascular, and other tissue compartments, including a variety of cutaneous sarcomas [2]. The epidemiology of these rare cancers, which include malignant adnexal tumors of the skin (MATSs), Merkel cell carcinoma (MCC), dermatofibrosarcoma protuberans (DFSP), atypical fibroxanthoma (AFX), pleomorphic dermal sarcoma (PDS), Kaposi sarcoma (KS), and leiomyosarcoma (LMS) cases, is less well documented [3,4]. Given their highly heterogeneous nature and rarity, significant gaps in our understanding of the etiology and clinical features of these cancers persist, hampering efforts to more reliably prevent or treat these tumors in an appropriately targeted manner.

Merkel cell carcinoma (MCC) is a rare neuroendocrine carcinoma of poorly understood cellular origin [5]. With a median age of diagnosis from 75 to 80 years and an incidence rate roughly 50-fold lower than that for melanoma at 0.3–1.6 per 100,000, MCC cases are most often characterized by solitary rapidly growing dermal/subcutaneous tumors in sun-exposed areas associated with mortality rates as high as 46% and a strong propensity for recurrence [5,6,7].

Cutaneous sarcomas are a heterogeneous group of rare tumors including AFX, PDS, DFSP, KS, and LMS that, in contrast to deep sarcomas, tend to have a relatively good prognosis [2,8]. These cutaneous sarcomas are also far rarer than cases of BCC, SCC, or melanoma, with handfuls of cases being reported across multi-year periods at many institutions [9,10]. Most commonly presenting on sun-exposed head and neck regions, AFX lesions exhibit varying histologic features, nonspecific immunohistochemical staining, and low-grade malignancy, comprising just 0.24% of treated skin cancers in one report [11]. PDS tumors share many histopathological features with AFX lesions but tend to exhibit deeper invasion and show a propensity to recur or metastasize such that both AFX and PDS cases have been proposed to lie along a spectrum of interrelated tumor phenotypes [12]. DFSP tumors are slow-growing intermediate malignancies of fibrohistiocytic origin with an estimated incidence of 4.2 per 1,000,000. Most commonly arising on the trunk, the drivers of DFSP oncogenesis are poorly understood, although prior trauma, surgery, or scarring are reported in approximately 10% of cases [13]. COL1A1-PDGFB fusion is detectable in virtually all cases of DFSP [14]. Patients tend to respond well to treatment, with reported 5- and 10-year recurrence-free survival rates of 86% and 76%, respectively, although local recurrence is a concern, emphasizing a need for appropriate treatment [15]. KS is a rare cutaneous sarcoma of endothelial origin that is among the most common cancers in patients living with HIV, with an age-standardized incidence rate of 0.39 per 100,000 persons globally in 2020 [16]. Sporadic cases of KS occur in HIV-negative patients, with certain ethnic groups at higher risk [17]. Cases of cutaneous LMS are very rare and account for only a fraction of the overall LMS disease burden, with a reported age-adjusted incidence rate of 0.6 per 1,000,000 person-years in the USA [10]. Risk factors for cutaneous LMS remain largely unknown, posing challenges to prevention, diagnosis, and treatment, although the prognosis tends to be favorable [18]. Rising incidence rates have been reported for MCC [5], with similar upward trends in rates of MATS [19] as well as AFX and PDS [20] diagnosis having been noted in recent years. These increases parallel an overall incline in the burden of skin cancer attributable at least in part to environmental and lifestyle factors, most notably chronic sun damage (CSD) stemming from exposure to UV light.

UV light exposure is perhaps the best-understood risk factor for many forms of skin cancer, inducing oxidative stress and characteristic patterns of DNA damage that are particularly closely associated with BCC and SCC incidence [21]. While it has been studied at length for BCC, SCC, and melanoma [22,23], knowledge of the relationship between UV exposure and the risk of particular rare skin cancer types remains somewhat more fragmentary. Many MATS subtypes are associated with UV exposure [24,25,26], which has also been firmly established as a risk factor for MCC, with solar radiation exposure and the depth of skin pigmentation, respectively, being positively and negatively correlated with the incidence of this rare neuroendocrine tumor type [5,27]. AFX and PDS are both strongly linked to UV exposure and CSD [28], with UV-related patterns of DNA damage having first been directly detected in the *TP53* gene in AFX patients 30 years ago [29]. Indeed, such recurrent patterns of UV-associated DNA damage have been described in many cutaneous sarcoma cases [30,31,32], with AFX and PDS cases reportedly exhibiting UV-induced mutational signatures 7a and 7b at similar rates in contrast to the predominance of signature 7a in melanoma [33]. UV-associated mutational signatures have also been reported in a subset of MCC cases [34]. DFSP cases, in contrast, harbor a distinct series of mutational signatures unrelated to UV exposure [35], consistent with their tendency to develop on the trunk rather than in sun-exposed areas.

Several other risk factors and molecular changes have been explored across rare skin cancer types. Notably, male predominance has been reported as a common finding in surveys of cutaneous sarcomas conducted to date [9], particularly for AFX, PDS, and KS cases. In analyses of larger patient cohorts, males reportedly comprised 72.6–86.2% of AFX patients [36,37]. Consistent with its strong similarity to and possible existence along a spectrum with AFX, PDS tends to be diagnosed at much higher rates among males as well. Both AFX and PDS are characterized by an extremely high tumor mutational burden (TMB), surpassing even that of SCC and melanoma cases [33,38], which may be attributable to chronic UV-associated DNA damage leading to the emergence of characteristic mutations in *TP53* and *CDKN2A/B* in many affected patients [39]. This high mutational load has clinical implications, as these cases may be better suited to immunotherapeutic treatment [38,40]. In other cutaneous sarcomas, however, TMB rates tend to be lower, as in DFSP [35], and only particularly aggressive cases of KS tend to exhibit a high TMB [41]. Specific mutations can also be used to guide differentiation among rare skin cancers, with *RB1* mutations primarily having been reported in cases of cutaneous LMS [42]. Distinct epigenetic alterations including changes in the hypermethylation of certain genes and patterns of histone methylation have been described for specific rare skin cancers [43,44], highlighting opportunities for therapeutic intervention that may be tied to particular inducing stimuli.

In addition to these environmental, genetic, and epigenetic drivers, other pathogenetic factors have been linked to the incidence of particular rare skin cancers. For example, genomic Merkel cell polyomavirus (MCPyV) integration is closely tied to the incidence of a subset of MCC cases [45]. Interestingly, the characteristic UV signature of DNA damage in MCC discussed above is only evident in patients with MCPyV− disease, such that the relationship between CSD and MCPyV+ MCC is less well understood [34,46]. The TMB of MCC tumors also varies as a function of MCPyV status such that MCPyV- cases tend to exhibit a higher mutational load [47]. Immunosuppression is another important contributing factor to rare skin cancer occurrence, as evidenced by the characteristically high rates of KS among HIV/AIDS patients prior to the advent of effective antiretrovirals capable of alleviating profound immunodeficiency [16]. MCC cases are similarly more common among HIV/AIDS patients and individuals with a history of hematological malignancies [5,48,49].

Our team has previously applied actinic elastosis (AE), a histopathological finding also referred to as solar elastosis characterized by the accumulation of abnormal amorphous or elastotic fibers and basophilic degeneration [50,51], as a biomarker for chronic UV exposure, which we have found differs in magnitude among subtypes of melanoma, cutaneous squamous cell carcinoma, and basal cell carcinoma cases [52,53]. Given the multifarious drivers of different rare skin cancers and our relatively incomplete understanding of the relative importance of these drivers, efforts to systematically explore biomarkers that can inform the prevention and/or treatment of these cancers are indicated. Accordingly, in this study, we extended our previously established method to rare skin cancers to investigate the differences between these tumors in more detail. Our main hypothesis was that AFX/PDS tend to have more peritumoral AE and are more likely to occur on the head and neck as a result of CSD, whereas DFSP tends to have less peritumoral AE and is more likely to occur on non-UV-exposed body sites.

## 2. Materials and Methods

### 2.1. Patient Characteristics and Inclusion Criteria

For the analysis of actinic elastosis (AE), we selected the most recent (*n* = 240) cases of AFX, PDS, DFSP, KS, MCC, and LMS at the University Medical Center of Regensburg spanning from 2014 to 2022. After screening, 30 cases had to be excluded (18 AFX, 4 PDS, 5 DFSP, 1 KS, 1 MCC, and 1 LMS) due to the poor quality of the histopathological specimen or the lack of peritumoral tissue to allow adequate AE grading. Clinical data, including the patient’s age at the time of the diagnosis, their sex, the body site, and information regarding immunosuppression (defined as prior or ongoing immunosuppressive medication use), were extracted using the i.s.h.med software 617 (IS-H 617) (Cerner Corporation, North Kansas City, MO, USA, run via SAP 6.0 software, SAP SE, Walldorf, Germany), which is used as the hospital management software at our institution. The face, head, neck, hands, and dorsal forearms were defined as “UV-exposed” body sites.

### 2.2. Histopathological Assessment

**Figure 1:**
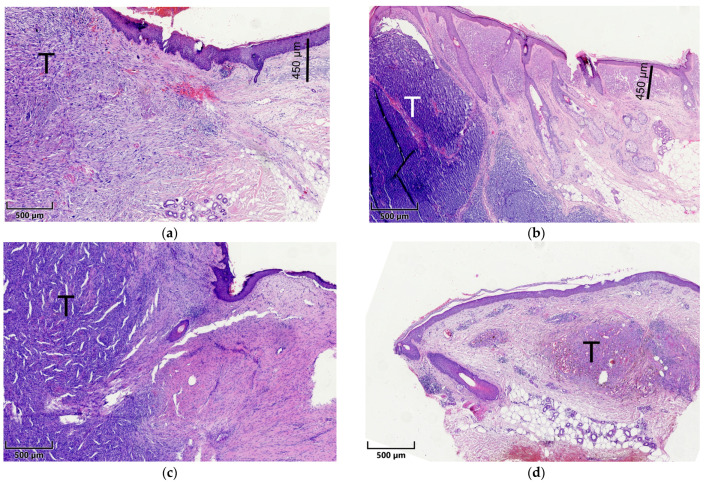
Exemplified histopathological assessment measurement of actinic elastosis and scoring (tumor-associated elastosis grading—TEG). The thickness/width of elastotic material was measured in the vicinity of the tumor (T) in the absence of tumoral stroma (scale bars). Elastosis was scored as follows: 0 = absent, 1 = low: less elastotic material than regular fibers (collagenous and elastic), 2 = moderate: more elastotic fibers than regular fibers, 3 = strong: complete or near complete loss of normal fibers/homogenous basophilic zone. (**a**) Pleomorphic dermal sarcoma; (**b**) Merkel cell carcinoma; (**c**) dermatofibrosarcoma protuberans; (**d**) Kaposi sarcoma.

Briefly summarized, tissues were embedded in paraffin after fixation and dehydration. Tissue sections of 4 µm were cut with a microtome and placed on microscopic slides. These slides were consecutively processed and stained with hematoxylin and eosin (H&E) as per standard protocol [54]. The histological examination was performed independently by two experienced dermatopathologists (K.D. and D.N.). The slides were sorted chronologically (date of excision) rather than by tumor type to ensure that the measurement was as unbiased as possible. To assess the depth of AE, the widest identifiable elastotic fiber or area of basophilic degeneration in the vicinity of the tumor and in the absence of tumoral stroma was measured orthogonally from the stratum granulosum using an ocular scale (Figure 1). To assess the degree of AE, a semi-quantitative score was established as follows: 0 = absent, 1 = low: less elastotic material than regular fibers (collagenous and elastic), 2 = moderate: more elastotic fibers than regular fibers, and 3 = strong: complete or almost complete loss of normal fibers or homogeneous basophilic zone. If these scores were consistent within a range of 1 point for the semi-quantitative score and a range of 20% for the AE depth measurement, the mean was calculated and used for subsequent analyses. The agreement between the two raters was moderate for the depth and degree of AE, indicating that the mean of the two raters was a good choice to obtain reliable values. Discrepant results were resolved using a discussion microscope. The depth was multiplied by the semi-quantitative score, which we defined as the tumor elastosis grading (TEG), as described previously [52,53]. Curettage material, punch biopsies, and specimens without surrounding normal tissue were excluded from further analyses. In the end, a total of 210 specimens from 210 patients were included in the statistical analysis.

### 2.3. Microscopy and Visual Illustration

Both dermatopathologists used an Olympus BX43 microscope (Olympus, Shinjuku, Japan) for their analyses. All photomicrographs were captured after slide scanning using a PreciPoint M8 microscope and scanner with ViewPointLight software version 1.0.0.9628 for imaging (PreciPoint GmbH, Freising, Germany); we refrained from digital enhancement. Figures were generated using IBM SPSS, version 25 (Armonk, NY, USA), and MS PowerPoint Professional Plus 2016, version 16.0.4266.1001 (Microsoft Corp., Redmond, WA, USA).

### 2.4. Statistical Analysis

Statistical tests were performed using IBM SPSS, version 25 (Armonk, NY, USA). The degree of AE and TEG for different tumor entities were compared using Student’s *t*-tests. Multiple regression and analysis of variance (ANOVA) approaches were used to evaluate the impact of clinical variables (age at the time of diagnosis and UV-exposed sites). The results were considered statistically significant at *p* ≤ 0.05.

## 3. Results

To assess the degree of AE in a meaningful and unbiased cohort of patients, we selected the 240 most recent specimens with a diagnosis of rare skin cancers from 2014 to 2022. After the exclusion of cases with poor-quality or missing clinical data, 81 AFX, 36 PDS, 27 DFSP, 20 KS, 27 MCC, and 19 LMS tumors from 210 patients were included in the analysis. The clinical variables of the study cohort are shown in Table 1. 

The mean ages at diagnosis for patients with these rare cancers were fairly similar, ranging from 63.9 to 70.9 years. In line with our hypothesis, AFX and PDS tumors were predominantly detected at UV-exposed sites (96% and 97.2%, respectively), whereas these rates were lower for the four other tumor types. Associations with anamnestic sunburns were less clear, with varied rates ranging from 33.3% for KS to 87.9% for PDS tumors. With the exception of KS patients, immunosuppression was relatively uncommon, affecting anywhere from 3.7% of DFSP patients to 25% of KS patients. AFX and PDS tumors primarily presented in the head and neck region, consistent with their relationship with CSD, while DFSP lesions were most commonly located on the trunk. KS was most commonly diagnosed on the extremities, and the localization patterns for MCC and LMS tumors were relatively evenly distributed across the head/neck, trunk, and extremities (Table 1).

**Figure 2:**
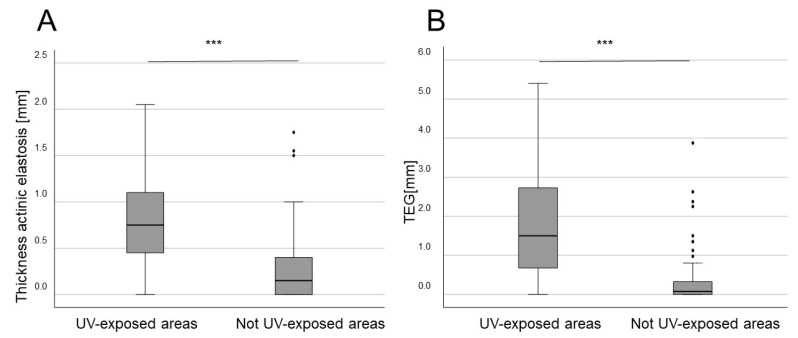
Mean thickness (depth) of AE and TEG correlated with UV-exposed body sites in all rare skin cancer entities combined. (**A**) The mean depth of AE across all groups stratified according to body site UV exposure (*** *p* < 0.001). (**B**) TEG (depth × semi-quantitative score) values for all groups stratified according to body site UV exposure (*** p < 0.001).

The depth and grading of AE were analyzed separately by two raters for all tumor types. Consistent with our previous data, when the data from these different cancers were pooled together, tumors arising in UV-exposed body sites exhibited higher average depth of AE and higher TEG values as compared to tumors in non-UV-exposed areas (both *p* < 0.001) (Figure 2). This was valid for both men and women.

**Figure 3:**
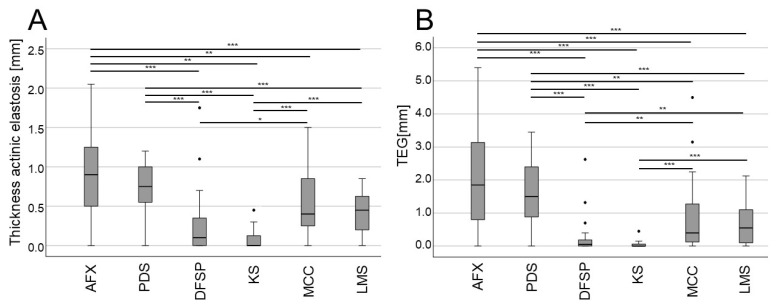
Mean thickness (depth) of AE and TEG, comparing different rare skin tumors. (**A**,**B**) Boxplots showing the mean depth of AE (**A**) and TEG (depth × semi-quantitative score) (**B**) for the indicated rare skin cancer types (* *p* < 0.05, ** *p* < 0.01, *** *p* < 0.001). Abbreviations: AFX, atypical fibroxanthoma; PDS, pleomorphic dermal sarcoma; DFSP, dermatofibrosarcoma protuberans; KS, Kaposi sarcoma; MCC, Merkel cell carcinoma; LMS, leiomyosarcoma.

**Figure 4:**
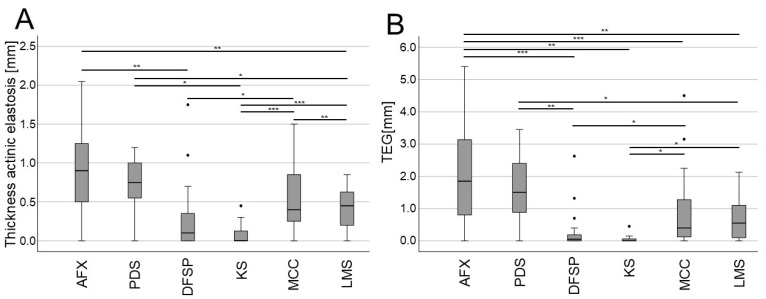
Evaluation of mean thickness (depth) of AE and TEG in different rare skin tumors when controlling for age and UV-exposed body sites. (**A**,**B**) Boxplots showing the mean depth of AE (**A**) and TEG (depth × semi-quantitative score) (**B**) for the indicated rare skin cancer types (* *p* < 0.05, ** *p* < 0.01, *** *p* < 0.001). Abbreviations: AFX, atypical fibroxanthoma; PDS, pleomorphic dermal sarcoma; DFSP, dermatofibrosarcoma protuberans; KS, Kaposi sarcoma; MCC, Merkel cell carcinoma; LMS, leiomyosarcoma.

When specifically analyzing the mean thickness of AE and TEG, significant differences were observed among tumor types (Figure 3). Notably, in line with their similarity to one another and close association with UV exposure, AFX and PDS tumors exhibited significantly greater AE thickness and TEG values as compared to most other tumor types, while DFSP and KS tended to exhibit the lowest AE thickness and TEG values (Figure 3). MCC tumors tended to exhibit greater AE thickness and TEG values as compared to DFSP and KS tumors, suggestive of a closer relationship with CSD. These differences in the depth of AE and TEG remained statistically significant or exhibited consistent trends even when age and UV-exposed body sites were controlled for through multiple regression analyses (Figure 4). 

## 4. Discussion

Our results highlight for the first time the value of AE thickness and TEG scores as surrogates for cumulative UV exposure when evaluating rare skin cancers, in line with the performance of these biomarkers noted in our prior studies focused on SCC, BCC, and melanoma cases [52,53]. When all cases included in this study were pooled according to whether or not the tumors arose on UV-exposed body sites, we found that AE thickness and TEG values were both significantly higher for sites that were classified as UV-exposed. This is an expected result that fits well with the current understanding of AE as a correlate for CSD [50]. However, no prior studies to our knowledge have systematically explored the AE profiles of cutaneous sarcomas or other rare skin cancers.

The most striking finding in this study was that when the six surveyed types of rare skin cancers were analyzed individually, AE thickness and TEG scores differed significantly among cancer types, with AFX/PDS tumors exhibiting significantly greater AE and TEG values even after correcting for age and UV-exposed body sites. This aligns with evidence that these cancers are closely associated with UV exposure and CSD, as supported by the presence of specific UV-related DNA damage signatures that partially overlap with those described in melanoma cases [29,30,31,32,33]. DFSP and KS cases, in contrast, exhibited AE thickness and TEG values significantly lower than those of other analyzed tumor types, consistent with their tendency to emerge on non-UV-exposed body sites in our cohort and their distinct mutational signatures not specifically related to UV irradiation [27]. The MCC and LMS cases included in our study cohort presented with intermediate phenotypes, with AE thickness and TEG values situated between those of AFX/PDS and DFSP/KS cases. UV exposure has been established as a risk factor for MCC [5,27], and some MCC cases present with UV-related DNA damage signatures [34], highlighting the relevance of further studies exploring the complex interplay between MCPyV status, CSD, and mutational outcomes in patients with this rare neuroendocrine tumor type. In contrast, we were unable to identify any strong evidence supporting a link between cutaneous LMS and sun exposure. As a majority of the LMS tumors in our study cohort developed on UV-exposed body sites and the overall case number was limited, additional studies will be essential to clarify the relationship between cutaneous LMS, CSD, and AE. 

As our newly established TEG scores were directly correlated with the age at the time of the diagnosis and UV-exposed body sites, coupled with their elevation in cancers known to be associated with UV exposure, we believe that AE analyses and TEG scoring offer value as a surrogate for CSD when evaluating rare cutaneous lesions.

Given the inherent rarity of the skin cancers included in this study and their reportedly increasing incidence rates [5,19,20], our case-related data spanning an 8-year period add valuable depth to the literature. The average ages at diagnosis for all patients included in this study were similar across tumor types. The majority of included AFX, PDS, and KS patients in this cohort were males, consistent with the male predominance for these tumor types described previously [9,36,37]. While the mean ages of AFX/PDS patients in our cohort were slightly below mean ages of 74–80 that have been reported previously [12,55], they were within the same general range. In contrast with prior reports [56], a majority of LMS cases in our study cohort were female, with this difference potentially being a consequence of our limited sample size. A greater proportion of KS patients were immunosuppressed as compared to patients with the other five tumor types, aligning well with the relationship between this otherwise rare cancer type and impaired immune function. The mean age of DFSP patients in our cohort was 63.9 years, which is notably higher than in other reports stating average ages between 30 and 40 years of age [57,58].

Our study has several limitations. First, as this was a single-center study, there may be some bias in terms of the specific characteristics of the presenting patient population and tumor diagnosis. However, the diagnoses were made by experienced dermatopathologists, most of whom were present at our institute throughout the study. Moreover, the University Hospital of Regensburg is the regional sarcoma center and diagnoses are independently verified by experienced soft tissue pathologists. Even so, future multicenter validation will be important to confirm the clinical relevance and generalizability of these results. Secondly, our study did not take other potentially relevant confounding factors such as occupational or recreational sun exposure habits, medication usage (e.g., antihypertensives), specific comorbidities, or history of other tumors into account, as these data were not sufficiently and consistently available from the patients’ medical records. There were also no available data regarding the ethnicity or skin type of these patients, although the vast majority of the patients treated in our center are either of Middle European or Eastern European descent. Moreover, we were also unable to establish the MCPyV status of MCC patients in this study. Given the previously described relationship between MCPyV status and UV-associated DNA damage signatures [5,46], MCPyV-specific subgroup analyses have the potential to add additional depth to our understanding of the AE features of MCC cases. Lastly, as noted in our prior studies using this approach [52,53], AE-based assessment of CSD lacks independent validation through immunohistochemistry or other techniques [59], underscoring opportunities for future research. Despite these limitations, our study represents an important step toward the more detailed histological comparison of different clinical subtypes of rare skin cancers, providing a valuable foundation for the more detailed interpretation of the role of UV exposure and other relevant risk factors in the onset of these malignancies.

## 5. Conclusions

In summary, our results demonstrate that AFX and PDS tumors are closely associated with higher levels of cumulative sun exposure, as determined based on AE thickness and TEG, while the same appears to be true to a lesser extent for MCC and LMS cases. KS and DFSP tumors, in contrast, did not exhibit increased AE thickness. In addition to serving as a source of demographic and clinical data related to these six skin cancer types, our findings highlight a new approach to analyzing these rare malignancies that may help inform their prevention, diagnosis, and/or treatment in the future. Future perspectives arising from our study include artificial intelligence (AI)-based automated analyses and their integration with clinicopathological parameters.

## Figures and Tables

**Table 1:** Clinical characteristics of the study population.

		AFX (n = 81)	PDS (n = 36)	DFSP (n = 27)	KS (n = 20)	MCC (n = 27)	LMS (n = 19)
Sex	Male	63 (77.8%)	31 (86.1%)	14 (51.9%)	17 (85%)	18 (66.7%)	4 (21.1%)
	Female	18 (22.2%)	5 (13.9%)	13 (48.1%)	3 (15%)	9 (33.3%)	15 (78.9%)
Age at diagnosis (y)		66.7 ± 17.2	70.9 ± 11.4	63.9 ± 17.1	66.1 ± 15.6	68.4 ± 17.5	66.2 ± 16.3
UV-exposed body site		95 (96%)	35 (97.2%)	5 (18.5%)	1 (5%)	10 (37%)	11 (57.9%)
Sunburns		52 (64.2%)	27 (75.0%)	12 (44.4%)	3 (15%)	15 (55.6%)	9 (47.4%)
Immunosuppression		10 (12.3%)	5 (13.9%)	1 (3.7%)	5 (25%)	3 (11.1%)	4 (21.1%)
Tumor Localization							
Head/Neck		77 (95.1%)	35 (97.2%)	2 (7.4%)	1 (5%)	8 (29.6)	10 (52.6%)
Trunk		2 (2.5%)	1 (2.8%)	18 (66.7%)	3 (15%)	8 (29.6%)	2 (10.5%)
Extremities		2 (2.5%)	-	7 (25.9%)	16 (80%)	11 (40.7%)	7 (36.8%)
Mean depth of AE (mm)		0.88 ± 0.52	0.73 ± 0.32	0.24 ± 0.4	0.56 ± 0.42	0.56 ± 0.42	0.42 ± 0.27
TEG (mm)		2.04 ± 1.4	1.61 ± 0.98	0.24 ± 0.55	0.88 ± 1.08	0.88 ± 1.08	0.67 ± 0.63

Abbreviations: AFX, atypical fibroxanthoma; PDS, pleomorphic dermal sarcoma; DFSP, dermatofibrosarcoma protuberans; KS, Kaposi sarcoma; MCC, Merkel cell carcinoma; LMS, leiomyosarcoma; UV, ultraviolet; AE, actinic elastosis; TEG, tumor elastosis grading.

## Data Availability

The data presented in this study are available from the corresponding author upon request.
